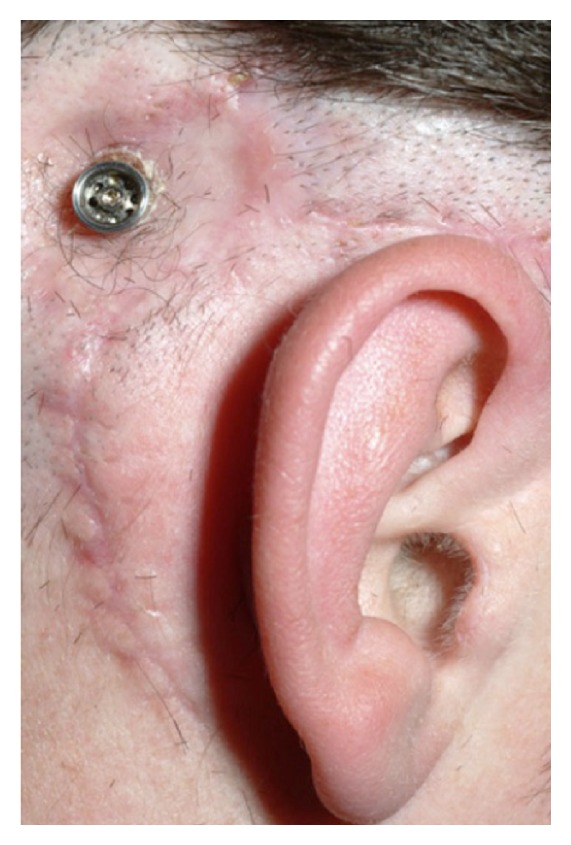# Single-Stage BAHA and Mastoid Obliteration

**DOI:** 10.1155/2012/765271

**Published:** 2012-10-10

**Authors:** Ajith George, Chris Coulson, Elizabeth Ross, Ranit De

**Affiliations:** ENT Department, Queen Elizabeth Hospital, Birmingham, B15 2WB, UK

## Abstract

A single-stage fitting of a bone-anchored hearing aid (BAHA) implant and abutment with mastoid obliteration both obviates the need for two separate procedures and utilises the BAHA soft tissue reduction in the mastoid obliteration. Such a procedure has good outcomes in terms of osseointegration and achieving a dry ear. We present a 6-patient case series report highlighting the technique of combined BAHA insertion and mastoid obliteration in six patients. All patients at twelve-month followup have a good degree of sound localisation and hearing thresholds with their BAHA and are free from the social stigma associated with a foul smelling discharging ear.

## 1. Introduction

Bone-anchored hearing aids (BAHAs) are an essential component of an ENT surgeons' armamentarium for treating patients with impaired hearing. Their indications include patients with conductive, sensorineural, and mixed ipsilateral losses and for contralateral stimulation in single-sided deafness. In selected groups, the satisfaction ratings are very high (98%) [1], with the number of nonusers low. 

The standard technique involves harvesting a split skin graft, centred on the point of optimal insertion 55 mm behind and 30 mm above the external auditory canal (EAC) [2]. Soft tissue reduction is then performed prior to inserting a titanium fixture (and abutment) and replacing the skin graft. There are multiple reasons for the soft tissue reduction: (a) to reduce signal loss and decay from mechanical energy being absorbed by the surrounding myofascial tissues from the titanium implant; (b) to achieve thin immobile around the abutment preventing the creation of granulation tissue at the skin BAHA junction as can happen with mobile skin; (c) to create hairless skin to enable easy use and cleaning of the BAHA; (d) to reduce skin height ensuring that the BAHA is easy to fit and the aid does not touch the skin reducing the energy imparted to the abutment. The tissue from the soft tissue reduction is routinely discarded. 

BAHAs can be used with great benefit in patients with conductive deafness, but it is in the rehabilitation of patients with active chronic ear disease where they out perform most other amplification devices as some patients develop otorrhoea when wearing a conventional hearing aid. This is due to a variety of factors, including reduced ventilation, increased humidity, and impairment of skin migration pathways. The otorrhoea is likely to be exacerbated in patients with mastoid cavities, not simply due to the above reasons, but also factors such as presence of a meatal stenosis, high facial ridge, deep sump, recurrent cholesteatoma, or exposed mucosa. In many such cases, surgery is the only option to provide a dry ear. The components of revision mastoid cavity surgery include the eradication of disease and granulation tissue, performing an appropriate meatoplasty, lowering the facial ridge, obliterating the cavity, and covering all exposed bone with fascia. Obliteration of the cavity leads to a smaller, shallower, less-troublesome cavity. Many techniques have been used for obliteration, including muscle flaps, bone dust, hydroxyapatite crystals, and cartilage. 

Successful mastoid obliteration with temporalis muscle or abdominal fat has been documented in conjunction with cochlear implantation previously [3]. The postauricular periosteal pericranial flap for mastoid obliteration has been shown to have up to a 90% success rate when looking at primary outcome measures such as the creation of a small dry low-maintenance cavity and secondary outcomes of haematoma, infection, flap necrosis, and meatal stenosis [4]. 

We present a method of combined BAHA and mastoid obliteration utilising the soft tissue from the BAHA soft tissue reduction to obliterate the cavity. This technique was used for good effect in six cases, for patients with a unilateral discharging mastoid cavity not amenable to medical treatment along with a conductive hearing loss. All patients were unable to use a conventional hearing aid due to otorrhoea. 

## 2. Case Series Presentation

### 2.1. Patients

Between May 2005 and May 2006, six patients underwent combined BAHA and mastoid obliteration. All six patients were male with an age range from 21 to 77 years. They all had primarily conductive deafness, some with an additional element of sensorineural deafness. Each patient had a persistently discharging mastoid cavity despite water avoidance and treatment with combined topical antibiotic and steroid drops and aural toilet for a minimum of 3 years prior to surgery.

### 2.2. Procedure

**Figure 1:**
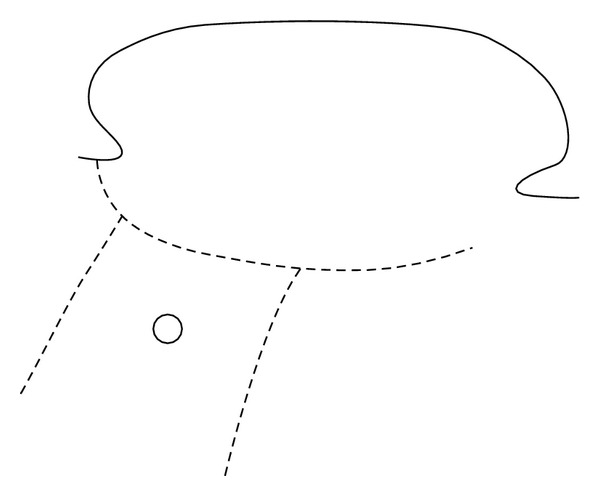


**Figure 2:**
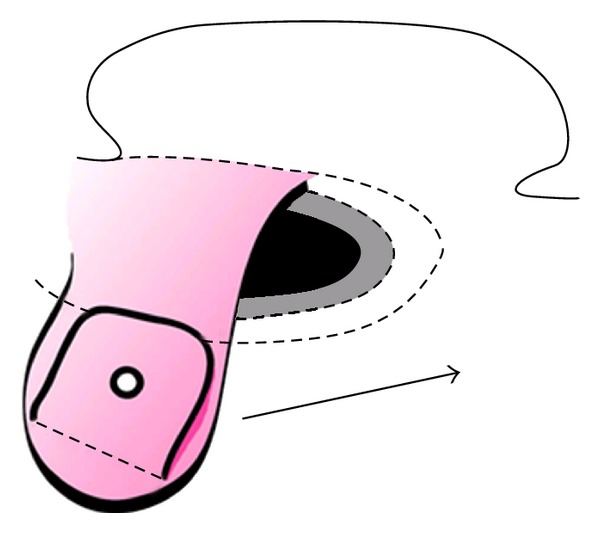


All operations were performed under a general anaesthetic. 2% lidocaine with 1 : 80,000 adrenaline local infiltration was administered at the site of the proposed incisions and in the ear canal. The postauricular incision, approximately 5 cms from the posterior aspect of the EAC incision, is designed to facilitate both procedures (Figure 1). Two posterior limbs are created, between which the split skin graft is elevated. Soft tissue reduction at the BAHA site is performed as a flap and left attached anteriorly as an anterosuperiorly based musculopericranial flap (Figure 2). All dermal and epidermal elements have to be removed, and then the flap is trimmed to an appropriate width which will fit inside the mastoid cavity. 

**Figure 3:**
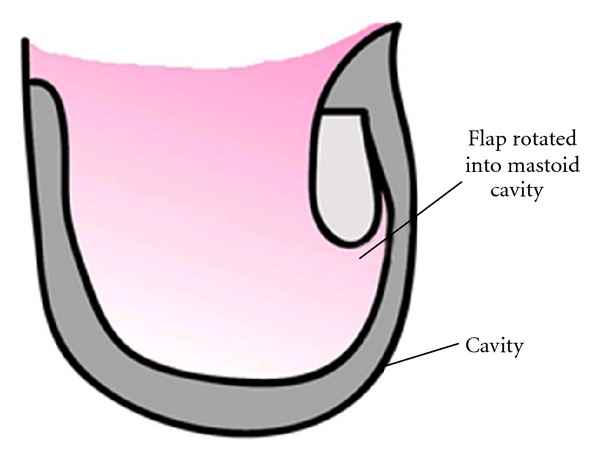


The mastoid cavity is revised, preserving as much of its healthy keratinous squamous epithelium as possible, which is then used to reline the revised obliterated cavity. The musculopericranial flap is narrower at its tip, compared to its base, and these dimensions are 10 mm and 25 mm respectively. The flap is now inverted into the revised cavity (Figure 3). Anchor sutures are applied from the base of the flap to sternomastoid in order to maintain its position inside the cavity until the ear has been closed and packed. Homologous bone pate and Tisseel are used to smooth over any gaps and irregularities, particularly spaces around the side of the flap and the obliterated cavity. The preserved mastoid keratinous epithelium is trimmed of any redundant or abnormal parts and placed over the flap, and a steroid- and antibiotic-soaked ribbon gauze pack is then inserted gently. The “clean procedure” (BAHA implantation) is performed first, and the “dirty procedure” (revision mastoidectomy) second. 

### 2.3. Results

All six patients were followed three months for a year. Osseointegration was achieved in all cases, and none have reported further ear discharge twelve months postoperatively. All patients are using their BAHA daily and report a vast improvement and good sound localisation.  Figure 4 demonstrates the postoperative appearance at 9 months.

## 3. Discussion 

A wide variety of methods have been described in the literature to obliterate mastoid cavities [5], and comparative analysis is difficult due to the lack in conformity of data collected. Pedicled musculopericranial flaps have been successfully used to perform mastoid obliterations [4, 6]. These have a dual benefit of eliminating dead space and promoting epithelialisation because of the vascular nature of the tissues. The multitude of axial and random flaps consisting of periosteum, temporoparietal fascia, temporalis fascia, and muscle would suggest that there is no ideal flap for mastoid obliteration. An anterosuperiorly based flap can be easily fashioned during surgery for a BAHA implant. Despite numerous previous operations the patient may have undergone, tissue for the flap is readily available. Performing both operations simultaneously is convenient for the patient and allows the surgeon to utilise the soft tissue reduction in the obliteration. 

Yoshida et al. [7] have reported good outcomes in patients with chronic otitis media with blind pit closure and BAHA. They have documented their technique in two separate stages as opposed to our single-stage approach. Closure of the external auditory canal eliminates exposure to potential pathogens and allows the patient to resume water exposure. However, there is a risk of implantation cholesteatoma, and patients need to be followed up annually with CT of MRI to exclude recurrence as direct microscopic evaluation is not possible. In theory, mastoid obliteration does carry a risk of the obliterating muscle flap covering residual cholesteatoma in the cavity. A good surgical technique will ensure that all squamous remnants are drilled out to reduce this possibility. Clinical observation can still be performed under the microscope looking for swelling under the muscle flap. 

The alternative to mastoid obliteration is cavity revision, including removal of recurrent disease, lowering the facial ridge, and performing a meatoplasty to aid suction clearance. The patient would still require outpatient aural toilet on a regular basis and would have to adopt water precautions to prevent active infection of the cavity. We therefore conclude that the single-stage procedure of BAHA insertion with mastoid obliteration using the soft tissue reduction has clear advantages over the other techniques discussed and should be considered in future practice. 

## Figures and Tables

**Figure 4:**